# Analysis of Frequency Stability and Thermoelastic Effects for Slotted Tuning Fork MEMS Resonators

**DOI:** 10.3390/s18072157

**Published:** 2018-07-04

**Authors:** Valentina Zega, Attilio Frangi, Andrea Guercilena, Gabriele Gattere

**Affiliations:** 1 Department of Civil and Environmental Engineering, Politecnico di Milano, 20133 Milano, Italy; valentina.zega@polimi.it (V.Z.); andrea.guercilena@mail.polimi.it (A.G.); 2 STMicroelectronics, AMG R&D, 20010 Cornaredo, Italy; gabriele.gattere@st.com

**Keywords:** MicroElectroMechanical Systems, resonators, modelling, optimization

## Abstract

MicroElectroMechanical Systems (MEMS) resonators are attracting increasing interest because of their smaller size and better integrability as opposed to their quartz counterparts. However, thermal drift of the natural frequency of silicon structures is one of the main issues that has hindered the development of MEMS resonators. Extensive investigations have addressed both the fabrication process (e.g., introducing heavy doping of the silicon) and the mechanical design (e.g., exploiting proper orientation of the device, slots, nonlinearities). In this work, starting from experimental data published in the literature, we show that a careful design can help reduce the thermal drift even when slots are inserted in the devices in order to decrease thermoelastic losses. A custom numerical code able to predict the dynamic behavior of MEMS resonators for different materials, orientations and doping levels is coupled with an evolutionary optimization algorithm and the possibility to find an optimal mechanical design is demonstrated on a tuning-fork resonator.

## 1. Introduction

Quartz crystals, thanks to their phase noise, thermal stability, ageing properties and power handling, were considered the frequency-reference industrial standard in the past century. Recently, MicroElectroMechanical Systems (MEMS) resonators (see e.g., [1]) entered the market (see [2]) of quartz oscillators as a possible solution to the increasing request of size reduction and integrability with the electronics and the other MEMS devices.

Several examples of MEMS resonators fabricated either in single-crystal silicon (see e.g., [3]) or polysilicon (see e.g., [4,5]) are available in the literature, but still need to be improved in terms of thermal drift and power handling (see [6]).

The thermal drift is mainly related to the intrinsic temperature dependence of the elastic constants (see e.g., [7,8,9,10]) and of the other thermal properties of silicon (i.e., thermal conductivity, specific heat and thermal expansion coefficient).

A strategy which led to encouraging results consists in modifying the structure of silicon through proper doping, either of *n*- or *p*- type. It has been recently proven that temperature stabilization with *n*-doping is applicable to various types of resonance modes and that second order temperature compensation comparable to that of quartz resonators is possible with doping higher than 10^20^ cm^−3^ (see e.g., [3,11]). Alternative approaches have been put forward in the literature such as temperature compensation methods that utilize either a tri-mode operation scheme (see [12]) or a nonlinear amplitude-frequency coupling (see [13]). Other solutions consist in the design of lateral micromechanical resonators supported by proper mechanical structures that introduce stresses to counteract temperature induced frequency shifts (see [14]), or of etch holes in Lamè resonators to modify their thermal drift (see [15]). Finally, active electronic compensations techniques are an alternative viable solution (see e.g., [16]).

As a model problem, we will focus on the classical single-ended tuning-fork (SETF) resonator of Figure 2a, fabricated in single-crystal silicon and vibrating according to an in-plane bending mode (see Figure 2b). It is worth stressing that very similar conclusions could be reached working with other types of resonators like torsional, Lamè or length extensional ones. This simple structure has the benefit of featuring virtually zero anchor losses [17], dissipation originating mainly from thermoelastic effects. We will also neglect any contribution from gas damping assuming near vacuum working pressures and sufficiently large gaps. However, if required, this dissipation could be estimated as suggested in [18,19].

In Section 2 the temperature dependence of all the mechanical and thermal properties of silicon is analyzed for different levels of doping and then applied to predict the frequency drift and the evolution of the quality factor of the SETF.

In Section 3, working on the analytical model of an idealized SETF, particular care is devoted to the investigation of the effect of material orientation. We show that the SETF, for a fixed level of doping, has an intrinsic lower bound of relative frequency drift associated with a specific material orientation and independent of the resonator dimensions.

However, the rather low thermoelastic quality factor is a strong limit for practical applications. A known strategy for improving *Q* consists in adding slots along the beams to reduce heat conduction (see [20,21,22,23]). Regrettably, slots may also sensibly increase the frequency drift in temperature. This topic, though of the greatest practical impact, has received relatively little attention in the literature and represents the main focus of this work. With this aim, in Section 4 we present a custom Finite Element Method (FEM) tool developed to compute the natural frequency and the quality factor of a MEMS resonator under different temperature conditions. After addressing some comparisons with the analytical solution, in Section 5 we introduce slots in the resonator beams and apply an optimization tool based on an evolutionary algorithm to obtain a device that shows good performance in terms of temperature stability and a high quality factor.

Closing remarks and future perspectives are reported in the last section.

## 2. Mechanical and Thermal Properties of Single-Crystal Silicon

Single-crystal silicon is a material with cubic symmetry (see [24]) and its stiffness matrix is defined by three elastic constants $c_{11}$, $c_{12}$ and $c_{44}$. If the Cartesian axes are aligned with the [100], [010] and [001] directions, it reads, for T = 25 °C:

(1) $$\left[C\right]=\left[\begin{array}{cccccc} c_{11} & c_{12} & c_{12} & 0 & 0 & 0\\ c_{12} & c_{11} & c_{12} & 0 & 0 & 0\\ c_{12} & c_{12} & c_{11} & 0 & 0 & 0\\ 0 & 0 & 0 & c_{44} & 0 & 0\\ 0 & 0 & 0 & 0 & c_{44} & 0\\ 0 & 0 & 0 & 0 & 0 & c_{44} \end{array}\right]=\left[\begin{array}{cccccc} 165.7 & 63.9 & 63.9 & 0 & 0 & 0\\ 63.9 & 165.7 & 63.9 & 0 & 0 & 0\\ 63.9 & 63.9 & 165.7 & 0 & 0 & 0\\ 0 & 0 & 0 & 79.6 & 0 & 0\\ 0 & 0 & 0 & 0 & 79.6 & 0\\ 0 & 0 & 0 & 0 & 0 & 79.6 \end{array}\right]\;\left[\mathrm{GPa}\right].$$

It is customary to express the temperature dependence of these coefficients with a quadratic expansion:

(2) $$c_{ij}=c_{ij}\left(@T\;=\;25{\;}^{\circ }C\right)\left(1+Tc_{{ij}_{1}}\Delta T+Tc_{{ij}_{2}}\Delta T^{2}\right)$$

with ij=11, 12 or 44 and ΔT the temperature shift with respect to the environmental temperature T = 25 °C. Limiting our attention to Posphorous doping, experimental data for $Tc_{{ij}_{1}}$ and $Tc_{{ij}_{2}}$ in (2) are available in many sources (see e.g., [8,9,25,26]).

These data have been tabulated (see Table 1) and interpolated in order to provide estimates also beyond the interval of available levels.

**Figure 1:**
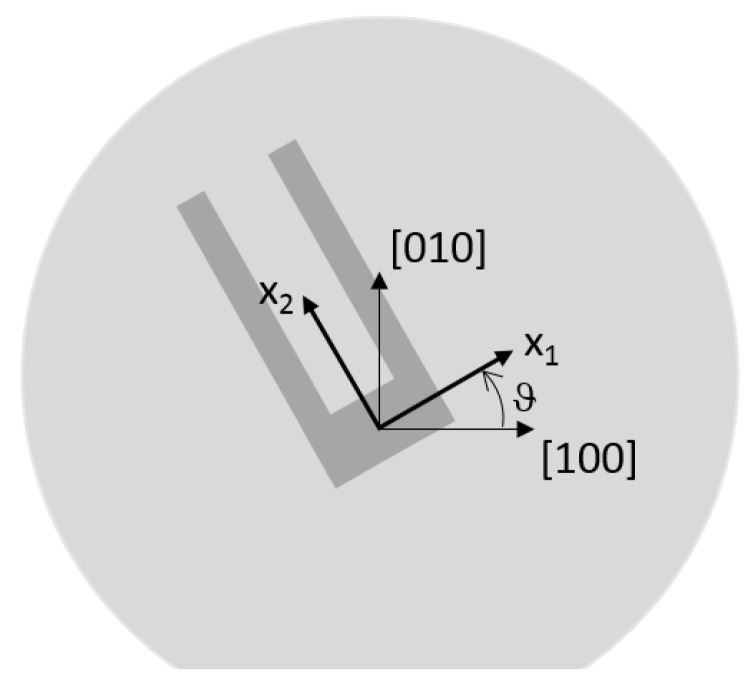
Material orientation of the local $x_{1},x_{2}$ axes with respect to the wafer [100] direction.

Once $c_{11},c_{12}$ and $c_{44}$ are computed for a given temperature and doping, the orientation ϑ of the mechanical structure with respect to the [100] direction (see Figure 1) is taken into account through a proper rotation applied to the stiffness matrix defined in (1). Please note that in the following, if not otherwise specified, the mechanical structure is designed in a reference frame aligned with the [100], [010] and [001] directions of the silicon wafer (i.e., ϑ = 0°) and the doping is Phosporous (P) with concentration 7.26 × 10^19^ cm^−3^. The elastic constants and their temperature dependences for such level of doping concentration are obtained by fitting the experimental results reported in Table 1. They read $c_{11}=161.41$ GPa, $c_{12}=66.13$ GPa, $c_{44}=78.56$ GPa, $Tc_{11_{1}}$ = −30.37 ppm/°C, $Tc_{11_{2}}$ = −81.30 ppb/°C^2^, $Tc_{12_{1}}$ = −133.86 ppm/°C, $Tc_{12_{2}}$ = −8.70 ppb/°C^2^, $Tc_{44_{1}}$ = −71.69 ppm/°C and $Tc_{44_{2}}$ = −30.39 ppb/°C^2^. Please note that, if not otherwise specified, only the data from [8] for the P-doping are used in the following for the sake of simplicity.

Thermal properties of silicon have been less investigated in the past as a function of doping. The thermal expansion coefficient (see [27]), the specific heat and the thermal conductivity (see [28]) are assumed doping-independent and equal to:

(3) $$\begin{array}{c} \alpha \left(T\right)=3.725\left(1-\exp\left(-5.88\times 10^{-3}\left(T-124\right)\right)\right)+5.548\times 10^{-4}T+0.0219)\times 10^{-6}\;\left[K^{-1}\right], \end{array}$$

(4) $$\begin{array}{c} c_{p}\left(T\right)=711+\frac{255\left({\left(T/300\right)}^{1.85}-1\right)}{{\left(T/300\right)}^{1.85}+255/700}\;\left[J/\left(\mathrm{Kg}\times K\right)\right], \end{array}$$

(5) $$\begin{array}{c} k\left(T\right)=145{\left(T/298\right)}^{-1.3}\;\left[W/\left(m\times K\right)\right], \end{array}$$

respectively. The temperature dependence of the silicon density is finally expressed as:

(6) $$\rho \left(T\right)=2330\left(1-3\alpha \left(T\right)\Delta T+9\alpha {\left(T\right)}^{2}\Delta T^{2}\right)\;\left[\mathrm{Kg}/m^{3}\right].$$

## 3. Analytical Model

**Figure 2:**
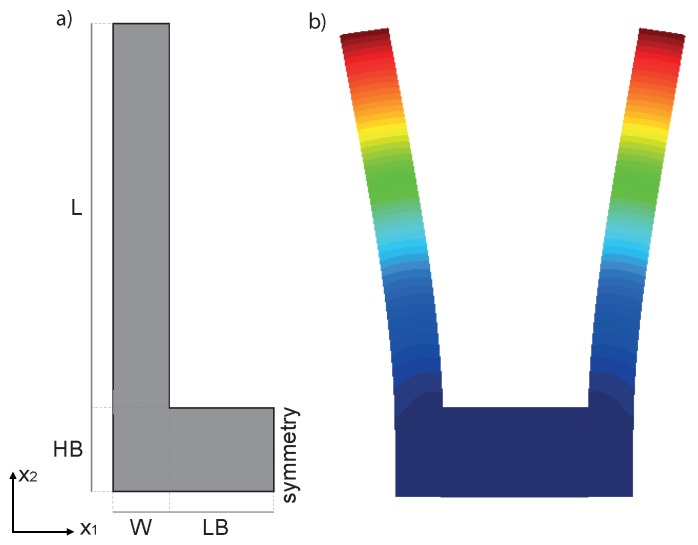
Tuning fork resonator. (**a**) Schematic view of the tuning fork resonator with out of plane thickness *t*. (**b**) First bending mode of the resonator. The contour of the displacement field is shown in color.

Applying the theory of slender beams to each vibrating arm in Figure 2 and neglecting the deformation of the lower connecting bar, the eigen-frequencies are:

(7) $$\begin{array}{c} f_{k}=\frac{\lambda_{k}^{2}}{2\pi }\sqrt{\frac{EJ}{\rho WtL^{4}}} \end{array}$$

where $\lambda_{k}$ are tabulated coefficients (e.g., $\lambda_{1}=1.875$), ρWt is the mass per unit length, $J=1/12tW^{3}$ is the inertia modulus for in-plane bending, *t* is the out-of-plane thickness and *E* is the Young modulus along the $x_{2}$ axis, computed as the 22 coefficient of the inverse ${\left[C\right]}^{-1}$ of the “rotated” stiffness matrix.

An estimate of the thermoelastic quality factor *Q* is given by the classical Zener’s formula (see [29]) for a single beam in bending:

(8) $$\begin{array}{c} Q=\frac{\rho c_{p}}{E\alpha^{2}T_{0}}\frac{1+{\left(\omega_{k}\tau_{z}\right)}^{2}}{\omega_{k}\tau_{z}}\;\mathrm{where}\;\tau_{z}=\frac{W^{2}}{\pi^{2}\left(k/\left(\rho c_{p}\right)\right)} \end{array}$$

where α is the thermal expansion coefficient, $c_{p}$ is the specific heat, *k* is the thermal conductivity, $T_{0}$ is the temperature and $\omega_{k}=2\pi f_{k}$ with $f_{k}$ defined in Equation (7).

### 3.1. Temperature Variation of Frequency

The temperature variation of the natural frequency of a MEMS tuning fork resonator is investigated in the reference temperature range [−35 °C–85 °C]. The temperature coefficient of frequency (TCf) is typically defined for a given temperature as:

(9) $$TCf=\frac{1}{f_{0}\left(@25{\;}^{\circ }C\right)}\frac{df_{0}}{dT}$$

but here a global measure of relative frequency variation in the temperature range, measured in part per million (ppm), is chosen as the indicator of the thermal drift of the device under study. It reads:

(10) $$\tilde{\Delta }f=\frac{\Delta f_{0}}{f_{0}\left(@25{\;}^{\circ }C\right)}\times 10^{6}\;\left[\mathrm{ppm}\right]$$

where

(11) $$\Delta f_{0}=\max_{T\in [-35{\;}^{\circ }C,\;+85{\;}^{\circ }C]}f_{0}\left(T\right)-\min_{T\in [-35{\;}^{\circ }C,\;+85{\;}^{\circ }C]}f_{0}\left(T\right).$$

In this section, we apply the analytical formula (7) to obtain an appoximate estimate of $\tilde{\Delta }f$ for the resonator depicted in Figure 2, having the dimensions specified in Table 2. A constant out-of-plane thickness t = 20 µm is considered.

**Figure 3:**
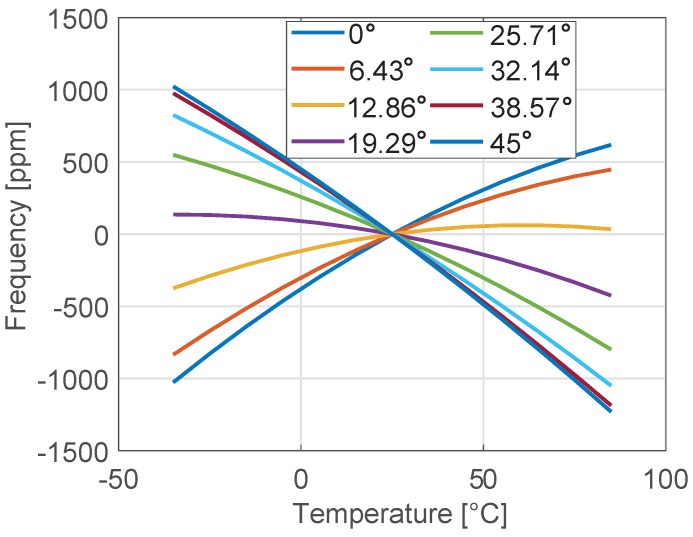
Frequency variation *f*_0_(*T*) − *f*_0_(25 °C) relative to *f*_0_(25 °C) for the tuning fork shown in Figure 2 for different orientations ϑ.

In Figure 3, the frequency variation (expressed in ppm) with respect to the value computed at 25 °C is reported for different orientations ϑ of the tuning-fork resonator with respect to the wafer (see Figure 1). A strong dependence of the thermal drift on ϑ (see e.g., [8]) is apparent: $\tilde{\Delta }f$ sweeps the entire range from 2100 ppm down to 160 ppm when ϑ increases from 0° to 45°. Due to symmetry, other values of ϑ would generate the same results.

**Figure 4:**
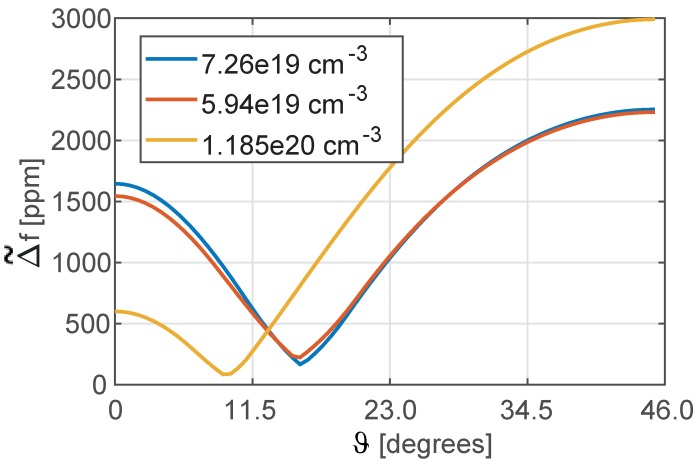
Maximum temperature variation of the natural frequency of the tuning fork in the range [−35 °C–85 °C] for different orientations of the device with respect to the silicon wafer and for different dopings of the silicon.

**Figure 5:**
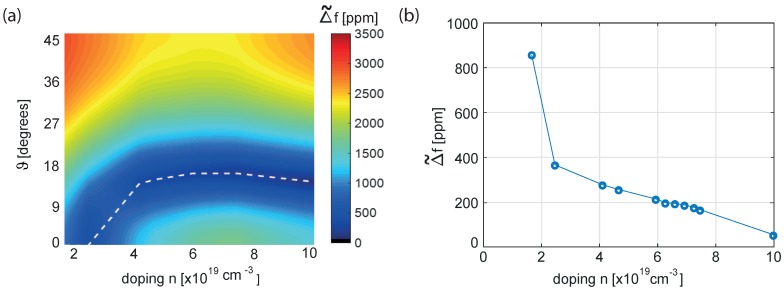
(**a**) $\tilde{\Delta }f$ for different orientations of the device with respect to the silicon wafer and for different *n*-dopings of the silicon. The white dotted line represents the minima of the contour plot. (**b**) Minimum temperature variation of the natural frequency of the resonator for different n-dopings of the silicon.

In Figure 4, the $\tilde{\Delta }f$ defined in (10) is reported for different levels of doping. Different minima are reached for each level: this confirms the already known result that the higher is the doping, the lower is the thermal drift of the MEMS resonator (see [9]). Moreover, each doping level is associated to a specific orientation ϑ of the mechanical structure that minimizes the $\tilde{\Delta }f$. In Figure 5a, the countour of $\tilde{\Delta }f$ is plotted for different orientations of the mechanical structure and for a variety of *n*-doping levels (not only Phosporous is considered for this analysis for the sake of completeness). In Figure 5b, a curve of minima is extrapolated from Figure 5a. Please note that in Figure 5, the elastic constants and their temperature coefficients for a set of *n*-doping levels (see dots in Figure 5b) have been taken either directly from the values reported in Table 1 for different *n*-dopants and from their fitting, and for this reason they may not be fully coherent being referred to different fabrication processes.

Moreover, it can be extrapolated that $\tilde{\Delta }f$ is expected to vanish for a *n*-doping slightly larger than 10^−20^ cm^−3^.

A remark is worth stressing here. Starting from formula (7) and Equations (2)–(5), it is possible to express the Young modulus *E*, the density ρ and the generic length *ℓ* as: $E=E_{0}\tilde{E}\left(T\right)$, $\rho =\rho_{0}\tilde{\rho }\left(T\right)$, $\ell =\ell_{0}\tilde{\ell }\left(T\right)$, respectively. Each variable is given by the product of its value at 25 °C by a suitable non dimensional (tilded) function of temperature. All dimensions scale as *ℓ*. Then,

(12) $$f=f_{0}\tilde{f}\left(T\right)\;\mathrm{with}\;\tilde{f}\left(T\right)=\frac{1}{\tilde{\ell (T)}}\sqrt{\frac{\tilde{E}\left(T\right)}{\tilde{\rho }\left(T\right)}}.$$

Next, applying the definition of $\tilde{\Delta }f$:

(13) $$\tilde{\Delta }f=\frac{1}{\tilde{f}\left(25{\;}^{\circ }C\right)}\left(\max_{\left[-35{\;}^{\circ }C,\;+85{\;}^{\circ }C\right]}\tilde{f}\left(T\right)-\min_{\left[-35{\;}^{\circ }C,\;+85{\;}^{\circ }C\right]}\tilde{f}\left(T\right)\right)$$

it is readily seen that $\tilde{\Delta }f$ is independent of the geometric dimensions.

### 3.2. Temperature Coefficient of Quality Factor

Since the dynamic behavior of a MEMS resonator strongly depends on the quality factor *Q*, in this section the focus will be on the study of such quantity. Please note that the stability in temperature of the quality factor is a key issue in MEMS resonators since it is related to the motional resistance of the device and consequently strongly influences the design of the control circuit.

The main contribution to damping in a MEMS tuning fork resonator is due to the thermoelastic effects. Gas damping (see [18]) is usually negligible since MEMS resonators are packaged in very low pressure conditions (see e.g., [2]). Also anchor losses (see [17]) can be neglected because of the chosen mechanical design that prevents the propagation of elastic waves through the anchors.

**Figure 6:**
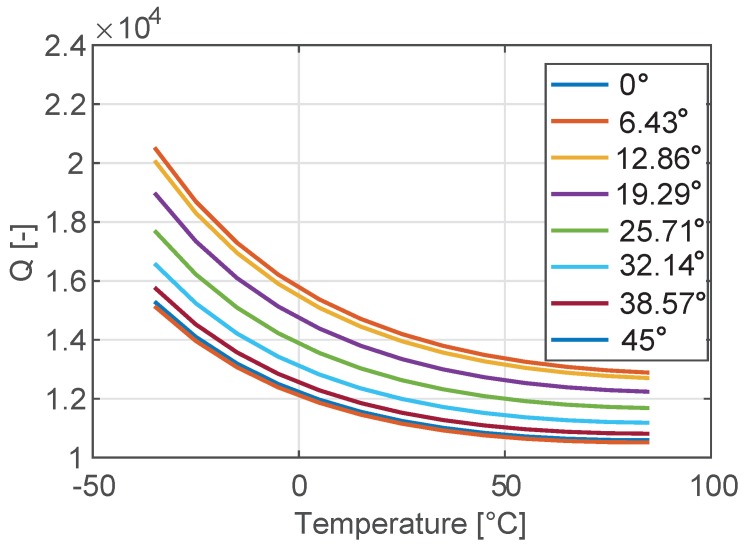
Temperature variation of the quality factor of the tuning fork in the range [−35 °C–85 °C] for different orientations ϑ of the device with respect to the silicon wafer.

In Figure 6 the temperature variation of the *Q* (see [29]) of the tuning fork resonator shown in Figure 2 is reported for different orientations of the mechanical structure. Please note that the *Q* is computed through Equation (8) under the hypotheses discussed in [29].

From Figure 6, it is evident that the dependence of the *Q* on the orientation of the resonator is not negligible, but it is at the same time not as important as for the thermal drift of the natural frequencies (see Figure 3).

Similarly, different levels of doping of the silicon do not influence the temperature dependence of the *Q* in a significant way.

## 4. Validation on the Real 3D Structure

Analytical formulas contain many simplifying assumptions among which we may cite 3D effects, rigid connecting bar, non perfectly 1D heat flow. Considering also the perspective of investigating structures with slots, we start applying a custom FEM code to compute the frequency and the thermoelastic *Q* of the MEMS addressed in the previous section under varying temperature conditions and arbitrary material parameters. The 2D geometry of the MEMS resonator shown in Figure 2 is meshed with quadratic triangular elements and is extruded in the out-of-plane direction.

The equations of fully coupled thermoelasticity assuming zero body forces read (see [30]):

(14) $$\begin{array}{cc} \rho \frac{\partial^{2}u}{\partial t^{2}} & =\mathrm{div}\sigma ,\;\sigma =d(\varepsilon -\alpha \left(T-T_{0}\right)1), \end{array}$$

(15) $$\begin{array}{cc} \rho c_{p}\frac{\partial T}{\partial t} & =\mathrm{div}\left(k\;\mathrm{grad}\;T\right)-\alpha T_{0}\frac{E}{1-2\nu }\frac{\partial \mathrm{tr}\varepsilon }{\partial t}. \end{array}$$

These equations are both enforced in a weak manner with a FE approach and the following strategy is applied. First, the mechanical mode of interest $u_{M}=f\left(x\right)e^{i\omega_{R}t}$ is computed from Equation (14) setting $T=T_{0}$, i.e., neglecting thermal coupling. The associated strain field is then inserted in Equation (15) to obtain the complex valued temperature $T=\left(\tau_{R}\left(x\right)+i\tau_{I}\left(x\right)\right)e^{i\omega_{R}t}$. Assuming the shape of the mechanical mode is not affected by thermal coupling, we set $u=f\left(x\right)e^{i(\omega_{R}+i\omega_{I})t}$ and compute $\omega_{I}$ from the full weak-form of Equation (14). Finally, the quality factor is obtained as $Q=\omega_{R}^{2}/\omega_{I}^{2}$.

**Figure 7:**
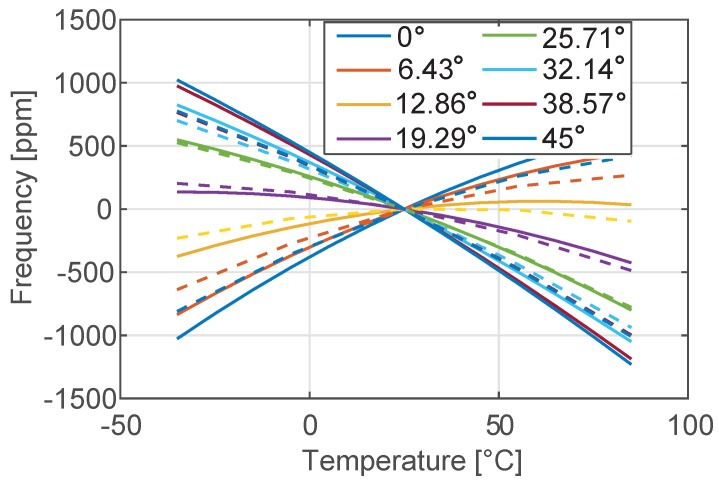
Temperature variation of the frequency of the tuning fork in the range [−35 °C–85 °C] for different orientations of the device with respect to the silicon wafer. Dotted lines denote numerical results, while continuous lines represent the analytical solution shown in Figure 3.

**Figure 8:**
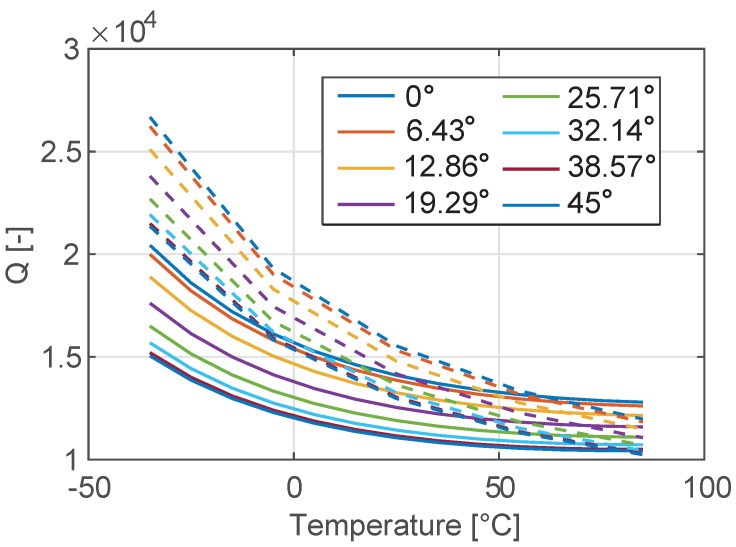
Temperature variation of the quality factor of the tuning fork in the range [−35 °C–85 °C] for different orientations of the device with respect to the silicon wafer. Dotted lines denote numerical results, while continuous lines represent the analytical solution shown in Figure 6.

The code is first utilized to reproduce the analytical plot of $\tilde{\Delta }f$ and *Q*. While formula (7) is known to be accurate, Equation (8) contains many approximations and larger deviations between numerical and analytical data are expected. This is confirmed in Figure 7 and Figure 8 where the comparison between the analytical predictions and the numerical results are reported in terms of temperature variation of both the natural frequency and the *Q*. As expected, the numerical results that take into account the full 3D geometry of the SETF differ from the analytical solutions more in terms of *Q* than of natural frequency. However, the qualitative conclusions drawn in the previous section remain unaffected:

- For a given level of doping and resonant mode type (e.g. bending-mode) the material orientation has a strong impact on $\tilde{\Delta }f$ and a clear minimum can be achieved. This value is essentially independent of the mode-order and geometric dimensions. The same minima are obtained analytically and numerically, although they might correspond to slightly different rotations of the material axes.
- The impact of material orientation on the *Q* value is minimal, and the rather low *Q* is an intrinsic limitation.

## 5. Optimization of the Tuning Fork Resonator

**Figure 9:**
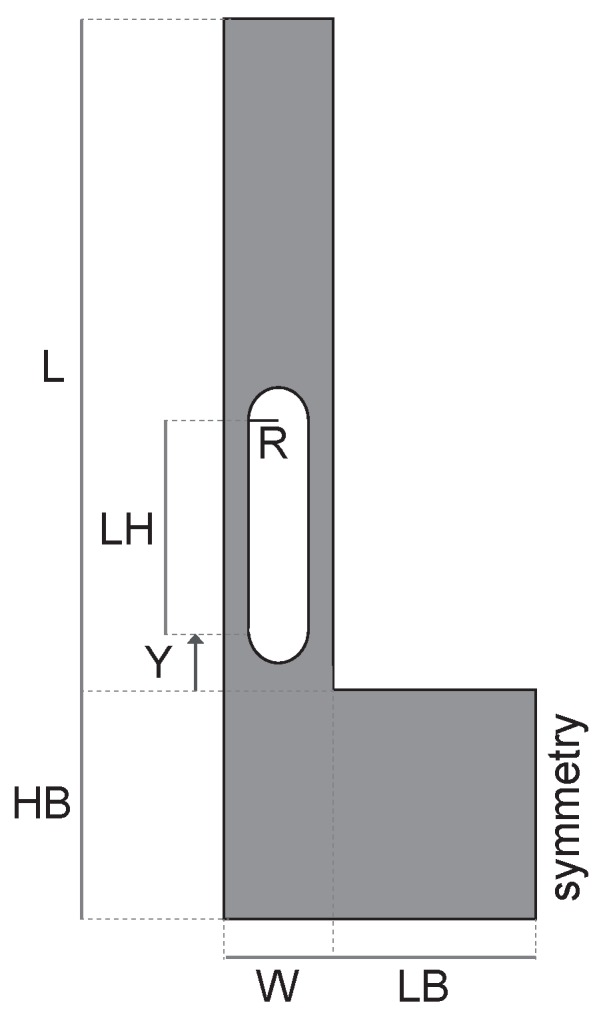
Tuning fork resonator with one hole: geometric dimensions defining the slit.

Having shown how to minimize the $\tilde{\Delta }f$ by adjusting the orientation of the resonator with respect to the silicon wafer and the doping level, the main goal of this section is to point-out a good strategy to maximize the quality factor of the MEMS resonator. Since it is known from the literature (see e.g., [20,21,22,23]) that slots may significantly reduce the thermoelastic damping, we now focus on the new model-geometry for a SETF depicted in Figure 9. Clearly, a multiplicity of slots could be included in the model, but the potentialities of the proposed approach are better put in evidence with a single one. Also, some constraints are included mimicking the technological process-dependent restrictions (i.e., R > 1 µm and the ones reported in Table 3 and Table 4). However, different constraints related to the specific fabrication process one wants to use, can be easily inserted in the procedure once fixed.

**Figure 10:**
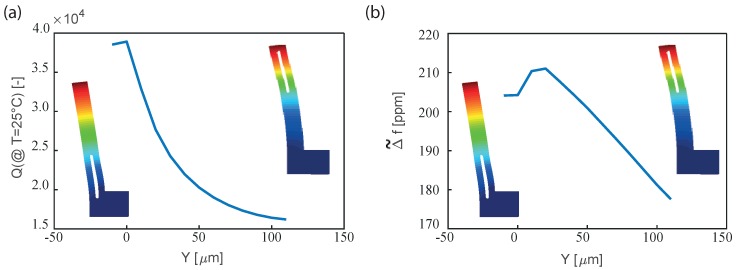
Influence of the hole position on the (**a**) quality factor and (**b**) on the variation of the frequency in the range [−35 °C–85 °C]: only the results for the orientation that minimize $\tilde{\Delta }f$ in the case of the SETF of Figure 2 is reported for the sake of clarity. In this analysis LH = 73 µm, R = 3 µm and the other geometric dimensions of Table 2 are employed.

As an example, and with reference to the dimensions of Table 2, in Figure 10a the quality factor computed numerically at 25 °C for different positions of the slots is reported, while in Figure 10b the corresponding frequency variations $\tilde{\Delta }f$ are shown. Please note that the position *Y* of the hole in the resonator influences both the quality factor and the $\tilde{\Delta }f$.

The mechanical design of the slots in a resonator is, as expected, a key point for the maximization of the quality factor while preserving a small $\tilde{\Delta }f$. An optimization procedure that takes into account all the geometric dimensions of the resonator is hence discussed in the next section.

### 5.1. Covariance Matrix Adaptation Evolution Strategy Optimization

The CMA-ES (Covariance Matrix Adaptation Evolution Strategy) is an evolutionary algorithm for non-linear non-convex black-box optimization problems in the continuous domain (see [31,32] for more details). The CMA-ES is a second order approach estimating a positive definite covariance matrix within an iterative procedure. At difference from quasi-Newton methods, the CMA-ES does not use exact or approximate gradients and does not even presume or require their existence. This makes the method applicable to non-separable and/or badly conditioned problems where gradient-based optimization algorithms usually fail.

The CMA-ES is only one possible choice in the family of non gradient-based algorithms. Alternative options are, for instance, Particle Swarm Optimization (PSO) (see e.g., [33]) or genetic algorithms (see e.g., [34]). It should be stressed, however, that our aim here is not to identify the best possible optimization procedure, but rather to prove a viable procedure for the improvement of resonators performance.

The CMA-ES does not require parameter tuning since finding the optimal parameters is considered to be a part of the algorithm design itself. Only the choice of the population size is left to the user: smaller sizes allow for faster convergence, larger sizes improve the global search performance (see [35] for more details). An initial solution, an initial standard deviation and, possibly, the termination criteria (e.g., a function tolerance) need to be set by the user as well.

The CMA-ES has been adopted as one of the standard tools for continuous optimization in many fields. Among the wide variety of applications of the CMA-ES optimization tool, one can mention the feedback control of combustion (see [36]), the turbulent friction drag reduction (see [37]), the design of a human-competitive lens system (see [38]) and the structural health monitoring (see [39]).

In this work, the CMA-ES is adopted for the optimization of the geometry of the SETF MEMS resonator in Figure 9. Depending on the quantity to minimize/maximize (e.g., quality factor, frequency variation in temperature), different objective functions and geometric constraints will be defined in the following. Eight optimization variables are considered:

(16) x=[YRLHLWLBϑHB]

where Y, R, LH, L, W, LB and HB are the geometric quantities shown in Figure 9 while ϑ defines the orientation of the resonator (see Figure 1): ϑ = 0° refers to the alignment of the $x_{1}$-axis with the [100] direction of the silicon wafer as previously stated.

Moreover, an initial value $x_{0}$ and a standard deviation σ for each of the eight variables are chosen: this implies that the CMA-ES starts the search at $x_{0}$ and initially performs the search mainly in the range ($x_{0}\pm 2\sigma$). In the following:

(17) σ=[101010101010510].

The population size is chosen according to the default option equal to (4 + floor(3 × log(8))) while the termination criteria, TolFun and TolX, are based on the changes of the objective function and of the optimization variables, respectively (e.g., TolFun < 1 × 10^−6^ means that the algorithm stops if changes of the objective function are smaller than 1 × 10^−6^). Lower and upper bounds are introduced in the optimization procedure in order to mimic feasibility criteria of the resonator (e.g., no negative dimensions and no slots radius smaller than 1 µm are allowed). Moreover, an upper bound for the in-plane thickness of the cantilever (i.e., W< 35 µm) is chosen in order to obtain a relatively small footprint of the MEMS resonator.

The doping P with concentration 7.26 × 10^19^ cm^−3^ and an out-of-plane thickness of the device equal to 20 µm are fixed. Please note that it is in principle possible to add such parameters in the optimization variables reported in Equation (16) without any further modification of the optimization procedure. A Matlab routine has been implemented in order to combine the CMA-ES algorithm with the FEM Fortran code already presented for the computation of the natural frequencies and the quality factor of the resonator. At each iteration of the optimization procedure, a new mesh is generated and the objective function is computed on the basis of the results of the FEM code.

#### 5.1.1. *Q* Maximization

As a first test, in this section the CMA-ES is applied to maximize the quality factor *Q* of the MEMS tuning-fork resonator shown in Figure 9. The reference environmental temperature is set to $T_{0}$ = 25 °C. The objective function of the CMA-ES in this case is chosen as:

(18) $$f_{obj}=-Q\left(@25{\;}^{\circ }C\right).$$

In Table 3, two optimal designs obtained through the CMA-ES for different constraints on the geometric parameters and on the natural frequency are reported. Both of them show a very high *Q*, but since no constraints have been imposed on the thermal drift of the natural frequencies, the $\tilde{\Delta }f$ is quite high (i.e., around 1000 ppm) with respect to the minimum found in the previous sections for the current doping level (i.e., 160 ppm). However, the results of Table 3 confirm the strong influence of the slots on the quality factor and offer a systematic procedure to design a MEMS resonator with low thermoelastic damping.

#### 5.1.2. Multi-Objective Function

Starting from the promising results in terms of *Q* maximization, here the objective function is properly chosen to combine the minimization of the frequency variation $\tilde{\Delta }f$ in the range of temperature [−35 °C–85 °C] and the maximization of the quality factor *Q* at 25 °C:

(19) $$f_{obj}=100\tilde{\Delta }f-Q\left(@25{\;}^{\circ }C\right)$$

where the weight 100 is only a reasonable proposal based on the results of the previous sections and could be indeed modified to overweight one of the two factors. In Table 4, the results of different optimization runs are reported. Please note that different constraints have been imposed on both the geometry and the natural frequencies of the resonators.

Some remarks are worth stressing: (i) the problem is highly non-linear and has several local minima that can be reached by varying initial conditions or constraints; (ii) a uniform convergence to the global minimum could be achieved by enlarging the population size at the cost of more intensive computations; (iii) independently of the previous remarks, all the different configurations summarized in Table 4 are near optimal from an engineering point of view, both in terms of quality factor and frequency variation. The minima for $\tilde{\Delta }f$ are in the order of those of Figure 4 for the same doping level while the *Q*(@25 °C) is consistently larger than the one shown in Figure 6. The CPU time for the results shown in Table 3 and Table 4 is around 48 hours of a computer DELL Intel Xeon CPU E3-1270 v3 @ 3.50 GHz with 32 GB of RAM. The use of the uncertainty quantification models (see e.g., [40]) that can replace the costly FEM analyses after a proper training is currently under investigation.

## 6. Conclusions

The dynamic behaviour of a SETF MEMS resonator has been thoroughly analyzed both with a simplified analytical model and a more comprehensive numerical tool. The dependence of the natural frequency and of the quality factor on the doping level of the silicon, on the orientation of the mechanical structure with respect to the wafer and on the geometry (e.g., slots) have been investigated in the temperature range [−35 °C–85 °C].

An optimization procedure based on the evolutionary algorithm CMA-ES (available for download in [41]) has been applied for the determination of the geometry of the MEMS resonator that maximizes a suitably chosen multi-objective function accounting both for the quality factor and the frequency drift in temperature. Different constraints have been imposed to prove the versatility and generality of the proposed approach.

This work introduces a powerful strategy for the design of MEMS resonators, since the objective function can be tuned or modified according to the needs of the users. The simulation approach can be further extended to include the simulation of fluid damping (see e.g., [18]) and of anchor losses (see e.g., [17]). These sources of damping, although negligible for the tuning-fork analyzed in the paper, could be of importance for different mechanical designs. An experimental validation of the proposed results is currently in progress.

## Figures and Tables

**Table 1:** Doping concentration dependence of the elastic constants of silicon and their temperature dependences. Elastic constants are expressed in GPa, while $Tc_{{ij}_{1}}$ in ppm/°C and $Tc_{{ij}_{2}}$ in ppb/°C^2^.

Doping Type	Concentration [cm^−3^]	*c*_11_	*c*_12_	*c*_44_	${\mathrm{Tc}}_{11_{1}}$	${\mathrm{Tc}}_{12_{1}}$	${\mathrm{Tc}}_{44_{1}}$	${\mathrm{Tc}}_{11_{2}}$	${\mathrm{Tc}}_{12_{2}}$	${\mathrm{Tc}}_{44_{2}}$
dop-*n*	3.00 × 10^13^ [26]	165.64	63.94	79.51	−63.4	−78.7	−55.4	−35	−56	−7
dop-*n*	1.98 × 10^19^ [26]	163.94	64.77	79.19	−39.2	−116.2	−58.7	−118	NaN	−28
P	4.10 × 10^19^ [8]	163	65.4	79.2	−34.5	−133.7	−67.8	−115	22	−51
P	4.66 × 10^19^ [8]	162.5	65.7	79.1	−32.5	−131.8	−68.7	−110	18	−43
P	6.60 × 10^19^ [9]	164	66.7	78.2	−34.2	−135.17	−67.8	−103.04	−1.1	−40.26
P	7.47 × 10^19^ [8]	161.4	66.1	78.5	−30.7	−134.9	−71.9	−78	−12	−31
As	1.20 × 10^19^ [9]	164.2	65.6	78.6	−46.58	−124.61	−63.12	−105.41	31.73	−45.21
As	1.66 × 10^19^ [8]	164	64.3	79.5	−48.5	−114.7	−63.7	−111	25	−58
As	2.46 × 10^19^ [8]	163.8	64.9	79.4	−44.2	−124.6	−65.1	−111	34	−55
Sb	1.30 × 10^18^ [9]	165.6	64.4	79.3	−65.5	−85.08	−60.92	−67.85	−28.1	−52.81

**Table 2:** Geometric dimensions of the tuning fork shown in Figure 2.

L	195 µm
HB	45 µm
W	20 µm
LB	34 µm
t	20 µm

**Table 3:** Optimal geometries computed through the CMA-ES optimization algorithm starting from the geometry shown in Figure 9. The employed objective function reads: *f_obj_* = −*Q*(@25 °C). All the geometric dimensions are reported in µm and the angles in degrees.

Geometry	Optimization Options	Results
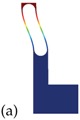	$x_{0}$ = [110 3 73 195 20 34 0 45] 0.3 MHz < $f_{0}$ < 0.7 MHz R < W/2–2.5 µm Y −R > −HB + 2.5 µm Y + LH + R < L −2.5 µm	x = [81.86 14.94 92.05 191.36 34.88 69.95 2.034 68.71] *f_obj_* = −*Q*(@25 °C) = −237831.19 $f_{0}$ = 0.30 MHz $\tilde{\Delta }f$ = 1115.21 ppm
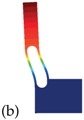	$x_{0}$ = [110 3 73 195 20 34 0 45] 0.4 MHz < $f_{0}$ < 0.6 MHz R < W/2–4 µm Y − R > −HB + 4 µm Y + LH + R < L −4 µm	x = [−7.27 10.37 64.18 155.44 28.75 66.82 0.09 69.11] *f_obj_* = −*Q*(@25 °C) = −82910.63 $f_{0}$ = 0.40 MHz $\tilde{\Delta }f$ = 936.86 ppm

**Table 4:** Optimal geometries computed through the CMA-ES optimization algorithm starting from the geometry shown in Figure 9. The objective function reads: $f_{obj}=100\tilde{\Delta }$*f* − *Q*(@25 °C). All the geometric dimensions are reported in µm and the angles in degrees.

Geometry	Optimization Options	Results
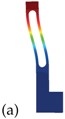	$x_{0}$ = [110 3 73 195 20 34 0 45] 0.3 MHz < $f_{0}$ < 0.7 MHz R < W/2–4.5 µm Y −R > −HB + 4 µm Y + LH + R < L −4 µm	x = [73.69 11.23 122.39 229.05 32.44 37.35 13.32 51.97] $f_{obj}\left(x\right)=-45416.74$ *Q*(@25 °C) = 62534.74 $f_{0}$ = 0.31 MHz $\tilde{\Delta }f$ = 171.18 ppm
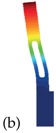	$x_{0}$ = [10 3 73 195 20 34 0 45] 0.3 MHz < $f_{0}$ < 0.7 MHz R < W/2–2.5 µm Y − R>−HB + 2.5 µm Y + LH + R < L −2.5 µm	x = [47.19 7.11 83.50 241.25 31.07 11.95 −12.996 93.35] $f_{obj}\left(x\right)=-12126.73$ *Q*(@25 °C) = 28164.73 $f_{0}$ = 0.45 MHz $\tilde{\Delta }f$ = 160.38 ppm
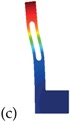	$x_{0}$ = [110 3 73 195 20 34 0 45] 0.4 MHz < $f_{0}$ < 0.6 MHz R < W/2–4.5 µm Y −R > −HB + 4 µm Y + LH + R < L −4 µm	x = [90.10 9.68 87.17 239.46 33.20 74.95 −12.834 60.95] $f_{obj}\left(x\right)=-14215.97$ *Q*(@25 °C) = 30955.97 $f_{0}$ = 0.44 MHz $\tilde{\Delta }f$ = 167.4 ppm
